# Annotations

**Published:** 1888-11-03

**Authors:** 


### Annotations.

A medical contemporary reports a case which possesses considerable interest for that large section of the community who use applications of different kinds for the head. The case was that of a man, thirty-seven years of age, who was troubled with what is called "scurfiness." He was in the habit of using for it a kind of "sulphur" ointment. The preparation consisted of five parts of wax, ten parts of sulphur, and about eighty-five of vaseline with a little oil of roses. This was applied every other day, and continued to be used for eight years. The ointment itself seemed perfectly harmless in composition, and its use for eight years without any apparent ill effects might have been considered sufficient to establish its character as an innocent medicament. But on a certain day in August the young man, without any premonitory warning, became suddenly giddy after eating his mid-day meal. His back and neck grew stiff, the face turned pale, and perspiration covered the forehead. Both the pupils were dilated, and would not react either to light or skin excitation. The head was pulled backwards and to the right by muscular contraction, and the muscles of the right half of the neck were hard and rigid. There was occipital headache, with nausea, and oppression of the chest. The pulse rose to 124 and became very small. The respiration was sixteen. The symptoms continued all day, and a restless night was passed. Afterwards the patient, by treatment, gradually recovered. It was elicited that he had suffered from headache for years. Dr. Eichbaam, who attended, considered that the cause of the symptoms was poisoning by sulphureted hydrogen, the gas being developed by the fat of the ointment and the sulphur, acted on by the warm skin. Small portions of gas he believed had constantly been absorbed by the skin of the scalp, and hence the result. Another case of the same kind is recorded in confirmation of these views. It is imperative that the public bear in mind the potency and danger of many of the drugs which are now in common use, both externally and internally. Numbers of substances which are harmless in the doctor's skilled and experienced hands, are highly dangerous when employed by those who know nothing either of their character or their effects. It may sometimes seem hard to have to call in the doctor for a trifle; but it is much better to do that than to run all kinds of risks, some of which may even involve life.

**Dangerous Ointments.**

It was hoped some time ago that the fashion of wearing the dead bodies of birds as trimmings for bonnets and hats was going out. Such a hope, apparently, is doomed to disappointment. Perhaps the day may come when people who have a little regard for such helpless creatures as birds will give them up to their fate. It really seems to be of no use to try to protect them. The loafer from the East-end of London goes forth with his cages and his lime and catches them. He, however, mostly retains the male. The other bird-murderer also goes forth on his cruel errand, and by preference catches and retains the female. He takes her in the nesting season, because the feathers are soft and beautiful then. What matters it to him that his victim is often the mother of a nest-full of helpless young, and that they are left in the nest to die of starvation; to die whilst piteously crying out hour after hour for the mother that never comes? The mother birds are killed, and the young left to die of starvation because certain women insist that it shall be so. Yet how gentle and sympathetic and tender those very women can *pretend* to be when it suits their convenience! How correct and nice is their taste in everything that relates to good manners! How shocked they are by vulgarity; how horrified by coarseness! If they could see themselves exactly as some men see them; could have it once driven in upon their consciences that in the estimation of all rational and right-feeling men they are incomparably inferior to many costermongers, crossing-sweepers, and untaught African negroes, they might for one moment pause and reflect upon their worthlessness. Is it really then come to this: That a nineteenth century woman is so utterly selfish, so hopelessly without brains or feeling, and so incapable of learning even the very elements of humanity that she must and will have birds to adorn herself with at whatever cost? At bottom it really is want of intellect. The idle modern woman of the wealthier classes is so self-indulgent, so pampered, and so spoilt that she can no longer be counted upon to exercise a reasoning faculty. Impulses, whims, and poutings alternate with fits of sulkiness or rage; and so she spends her life. When a man comes to the conclusion that any of his fellow-beings are either helplessly stupid or hopelessly unfeeling, he has still one source of consolation left: he knows they cannot live for ever. They will die some day. Whether that great change will open their eyes, or whether it will close them for ever who shall say? So far as their intellectual value is concerned the answer is probably of little or no moment. The movement in favour of the emancipation of women, it may be hoped, will not only give enlargement, but a sense of responsibility and duty. No man can contemplate without the deepest anxiety the gradually increasing mental weakness among the prosperous. If the stern necessities of the poorer class of ladies develop in them true strength of mind and sternness of moral fibre, most people will think poverty and necessity are blessings, though in disguise. Hardly any price is too great to pay for brains and a moral faculty.

**Women and Their Victims.**

The vagaries of a certain class of young women are legion. The particular amusement which affords some of them the most exquisite pleasure seems to be that of tormenting and tyrannising over the other sex. It is well known that the effect of the tender passion upon young men of peculiar susceptibility is often to deprive them of all self-respect and to reduce them to the level of abject slaves. A young man of this class will do and say the most ridiculous things if only the lady of his choice bids him. She may refuse his offers with scorn, but the sole effect of her refusal is to make him go on repeating them. One such Lothario the writer knows of, who proposed twelve times, and twelve times the proud madam said "No." Once more, however, he tried his fortune, and the thirteenth proposal was successful. Some engaged women consider that the chief pleasure of being engaged is to show their power over the men of their choice. Others will indulge in the most barefaced flirtation for no other reason but to provoke a lover to jealousy and anger. A case of the former kind was brought to light in the Glasgow breach of promise court the other day. Miss Maggie Lockhart Watson had for a lover Mr. William Kirkland. Mr. Kirkland, it appears, was fond of a cigar, but Miss Watson was not. She not only objected to cigars herself, but was determined that Mr. Kirkland should object to them too. She probably thought that as it was her intention to keep him in his place after marriage, she had better begin as she meant to go on. She therefore delivered to him an ultimatum on the cigar question. The formula was as brief as it was business-like, and in Miss Watson's opinion could produce but one result. "You must choose between me and a cigar," wrote this categorical exponent of the rules of a love engagement. Mr. Kirkland made his choice, but he did not choose Miss Watson. Then the lady changed her tune. It is not stated that she urged him to marry her, but she did bring him into the breach of promise court because he failed to do so. The Sheriff, however, sympathised with Mr. Kirkland, as we do. The moral to be pointed is that young women should show towards their lovers exactly the same degree of consideration as they wish their lovers to show to them. Spoilt children are bad enough, but spoilt women are unendurable.

